# Recurrent cerebellar ischemic infarctions and stereotyped peri-ictal sympathetic responses in a near-SUDEP patient with cardiovascular risk factors

**DOI:** 10.1016/j.ebr.2023.100605

**Published:** 2023-05-11

**Authors:** J.L. Vega, A. Carrasco, N. Karim, M. Stewart, W. Bell

**Affiliations:** a East Carolina University Medical Center, Greenville, NC, United States; b TeleNeurologia SAS, Medellin, Colombia; c Brody School of Medicine, East Carolina University, Greenville, NC, United States; d Departments of Neurology, State University of New York Health Sciences University, Brooklyn, NY, United States; e Physiology and Pharmacology, State University of New York Health Sciences University, Brooklyn, NY, United States

**Keywords:** near-SUDEP, Postconvulsive leukocytosis, Neurogenic pulmonary edema, Cerebellar ischemic infarction, Catecholamines

## Abstract

We report a 60-year-old woman who presented to the emergency department after experiencing a witnessed unknown onset bilateral tonic clonic seizure (GTCS) that culminated in cardiac arrest. A neurology consultant uncovered a years-long history of frequent episodic staring followed by confusion and expressive aphasia, which strongly suggested that she suffered from epilepsy. Thus, her cardiac arrest and subsequent resuscitation met criteria for a near-sudden unexpected death in epilepsy (SUDEP) diagnosis. Serial bloodwork demonstrated transient troponin I elevations and leukocytoses, while a brain MRI revealed global cerebral anoxic injury and a small acute right cerebellar ischemic infarction. A review of her medical record uncovered a hospitalization sixteen months earlier for a likely GTCS whose workup showed similar troponin I elevations and leukocytoses, and surprisingly, a different small acute right cerebellar ischemic infarction in the same vascular territory. To our knowledge, this is the first report of subcortical ischemic infarctions occurring concurrently with GTCSs in a near-SUDEP patient. Aside from illustrating the key role of inpatient neurologists in the diagnosis of near-SUDEP, this manuscript discusses the potential significance of postictal ischemic infarctions, transient asymptomatic troponin elevations, and transient non-infectious leukocytoses in epilepsy patients with cardiovascular risk factors.

## Highlights

• Troponin and leukocyte elevations could signal SUDEP risk in some epilepsy patients.
• Inpatient neurologists may be necessary to discern near-SUDEP cases from sudden cardiac arrests.
• Epileptic convulsions may exhibit patient-specific stereotypical sympathetic responses.
• SUDEP pathophysiology may affect the posterior cerebral vasculature.

## Introduction

A sudden unexpected death in epilepsy (SUDEP) diagnosis is typically assigned postmortem when a patient with known epilepsy dies suddenly and unexpectedly following one or more epileptic seizures and there are no alternative explanations, such as trauma, drowning, status epilepticus, or myocardial infarction, to explain his or her demise.[1] Most inpatient physicians are unaware of SUDEP, not only because of its retrospective and complex diagnostic criteria, but also because SUDEP victims are typically brought to the attention of pathologists, not clinicians. Yet, a diagnosis of “near-SUDEP,”[1] also called “resuscitated SUDEP,”[2] should be given when epilepsy patients who experience cardiac arrest immediately after a seizure are resuscitated and survive at least one hour. As this clinical scenario invariably requires intensive care unit (ICU) management, inpatient physicians are probably exposed to near-SUDEP victims much more frequently than they might expect. A timely recognition of such patients not only allows for an accurate diagnosis, but also presents a unique opportunity to investigate the pathophysiological clues that terminal seizures[3] might leave behind on imaging studies and blood tests. The present manuscript describes a patient with a history of cardiovascular disease who experienced a cardiac arrest immediately after a witnessed unknown onset bilateral tonic clonic seizure (GTCS). A near-SUDEP diagnosis was not reached until a neurology consultant uncovered what amounted to a history of untreated epilepsy. The subsequent workup and chart review suggest that the patient’s cardiac arrest was precipitated by a combination of cardiovascular risk factors and a semiological, or stereotyped, *peri*-ictal sympathetic response.

## Case presentation

**Fig. 1:**
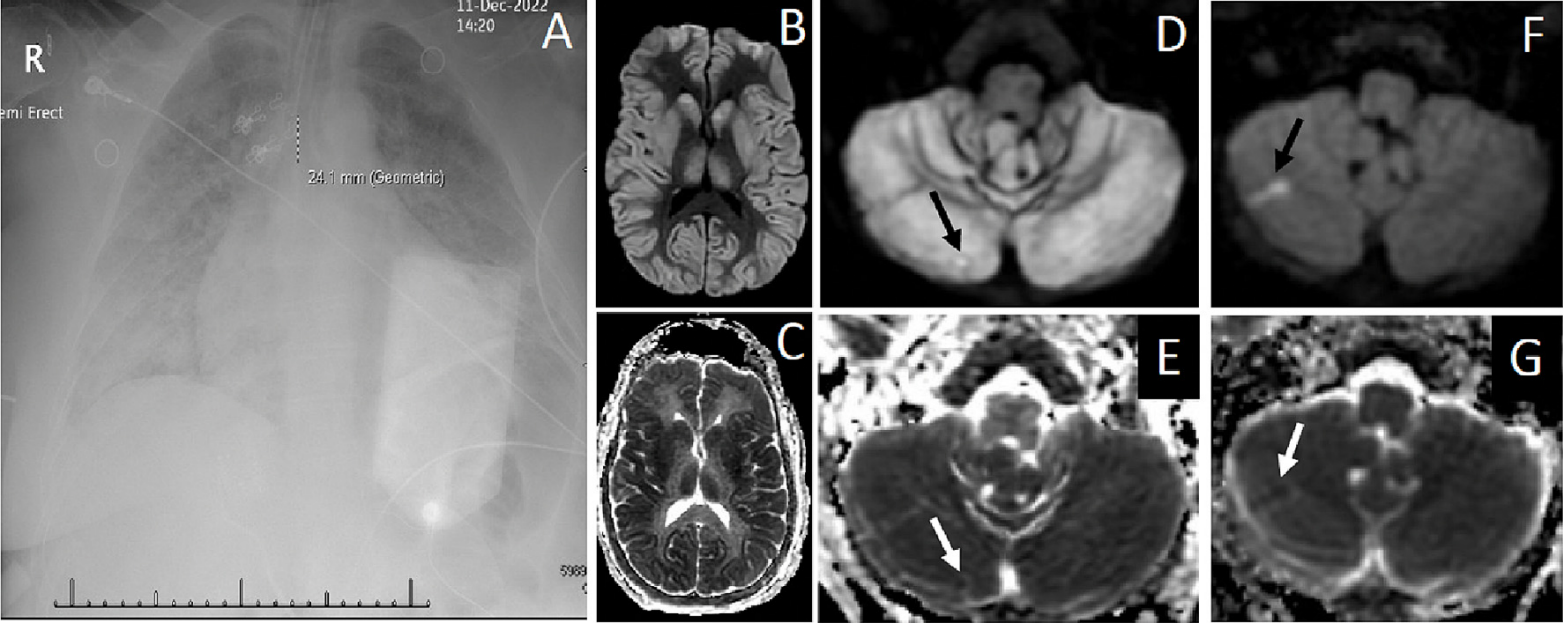
Neurogenic pulmonary edema, anoxic brain injury and recurrent ischemic infarctions in a near SUDEP patient. **A**: Chest X-ray taken shortly after ED arrival following a witnessed GTCS that culminated in cardiac arrest. The image shows florid bilateral postictal neurogenic pulmonary edema. The letter R in the X-ray image refers to the patient’s right side. **B-G**: Diffusion weighted imaging and apparent diffusion coefficient correlates demonstrating diffuse anoxic brain injury (**B-C**) and two small cerebellar ischemic infarctions, one diagnosed during the patient’s near-SUDEP hospitalization (**D-E**) and one during an earlier hospitalization, sixteen months earlier, for a presumed GTCS (**F-G**). Note the adjacent location of the two ischemic infarctions within the same cerebellar vascular territory. Black and white arrows show the ischemic infarctions’ DWI and ADC signals, respectively.

**Fig. 2:**
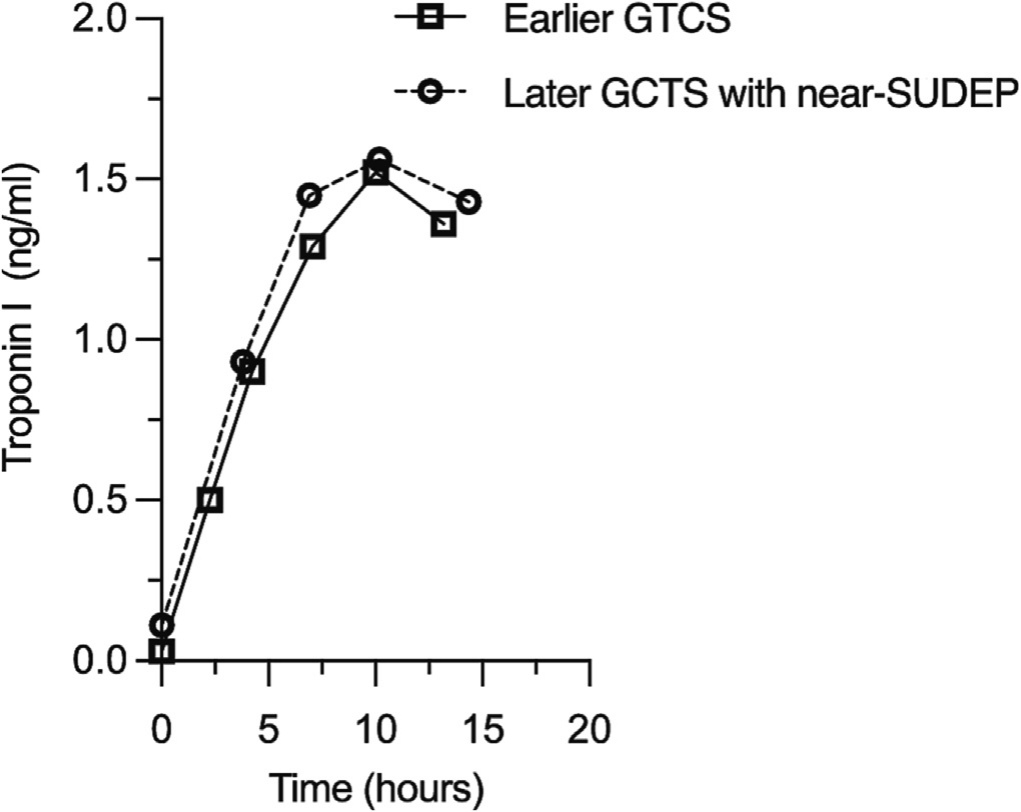
Serial troponin I levels obtained in the emergency department after a GTCS sixteen months before (squares) the GCTS that resulted in near-SUDEP (circles). The time points on the X axis represent times elapsed after the earliest troponin was drawn (time zero) on each of the two admissions.

A 60-year-old woman with a past medical history of paroxysmal atrial fibrillation (PAF), hypertension, hyperlipidemia, and obesity, (BMI 40.5 kg/m^2^) but no known history of sleep apnea suffered a GTCS during which copious pink froth dripped from her mouth. A close friend who had spent the morning with her, and who had witnessed the seizure, activated emergency medical services (EMS) and reported that she had acted normally until the onset of her seizure. Upon arrival, emergency medical technicians (EMTs) observed postictal combativeness followed by cyanosis, and by a pulseless electrical activity (PEA) cardiac arrest that required five minutes of cardiopulmonary resuscitation (CPR) until the return of spontaneous circulation (ROSC). Because an attempt to intubate in the field failed, she was ventilated via bag-valve mask during transport to the ED where endotracheal intubation was successful. Soon after, she experienced a ventricular fibrillation cardiac arrest which transitioned to PEA and required six minutes of CPR, defibrillation, and two rounds of intravenous epinephrine until ROSC. She was started on vasopressors and transferred to the ICU. Her physical examination at that point demonstrated cyanosis, diffuse rhonchi and wheezes on auscultation and complete unresponsiveness to voice and pain. Her pupils were equal but non-reactive to light and her tone was flaccid. The initial chest X-ray showed severe bilateral pulmonary edema (Fig. 1A), while serial bloodwork showed transient elevations of both troponin I (Fig. 2) and peripheral leukocytes (Table 1.). Collateral information obtained by a neurology consultant on the second hospital day uncovered a years-long history of frequent stereotyped episodes of unresponsive staring followed by several minutes of confusion and difficulty speaking. She had never sought medical attention for these episodes, which had become more frequent in recent months. A brain MRI performed four days after admission revealed global cerebral anoxic injury (Fig. 1 B-C) and a punctate right cerebellar ischemic infarction (Fig. 1 D-E). Faced with a dismal prognosis, her family opted to withdraw artificial life sustaining measures. An autopsy was not performed.

**Table 1:** Relevant data obtained during the patient’s two admissions, one for a witnessed generalized tonic clonic seizure (GTCS) resulting in a near-SUDEP diagnosis and the other for a presumed GTCS, sixteen months earlier. The timeline columns provide exact post-admission days and times when tests were performed. ABG = arterial blood gas; BP = blood pressure; BUN = blood urea nitrogen; bpm = beats per minute; BMP1 and BMP2 = first and second basic metabolic panels collected during each admission, respectively (reference ranges: Na = 136–145 mEq/L, K = 3.4–4.4 mEq/L; Cl = 98–107 mEq/L; CO2 = 23–31 mEq/L; Glu = 70–105 mEq/L; BUN = 10–20 mg/dL; Cr = 0.57–1.11 mg/dL); CXR = chest x-ray; CBC1 and CBC2 = first and second complete blood counts collected during the admission, respectively (reference ranges: WBC = white blood cells: 4.5–11.0 10^9^/L; Hb = hemoglobin: 12.0–16.0 g/dL; Ptl = platelets: 150–440 10^9^/L; % PMN = polymorphonucleocytes; 40–60%; Lymph = lymphocytes = 20–40%); cEEG = continuous EEG; D1-D3 = day 1–3 post-admission; EF = ejection fraction; FIO2 = fraction of inspired oxygen; HR = heart rate; HTN = hypertension; IVC = inferior vena cava; MPTA = medications prior to admission; ND = not done; PFO = patent foramen ovale; PMH = past medical history; ROSC = return of spontaneous circulation; SpO2 = hemoglobin oxygen saturation; T = temperature; OU = otherwise unremarkable.

**Timeline**	**Near-SUDEP admission**	**Timeline**	**GTCS admission (sixteen months earlier)**
D1 14:03	ROSC; HR 123 bpm, BP 138/71, SpO2 68% (FIO2 65%) T 34.1^o^ C	D1 13:36	HR 116 bpm, BP 177/85, SpO2 94% (FIO2 21%) T ND
D1 14:06	EKG: Sinus tachycardia at 127 bpm; OU	D1 14:40	EKG: Sinus tachycardia at 99 bpm; OU
D2 06:06	2D Echocardiogram: EF 60–65%; mildly dilated IVC; OU	D2 06:26	2D Echocardiogram: EF 55–60%; severely dilated left atrium; No PFO; OU
D1 14:27	ABG pH 6.62, pCO2 114 mmHg, pO2 107 mmHg, HCO3 12 mEq/L		ABG: ND
D1 14:37	CXR: severe pulmonary edema	D1 21:47	CXR: Clear lungs
D1 14:15	CBC1: WBC **12.52** Hb 11.6 Ptl 365 PMN 57% Lymph 43%	D1 14:36	CBC1: WBC **15.53**; Hb9.3; Ptl 325; PMN 90% Lymph 10%
D1 16:31	CBC2: WBC **10.51**; Hb 8.8 Ptl 252 PMN 78% Lymph 22%	D2 04:57	CBC2: WBC **12.87**; Hb9.6; Ptl 330; PMN 85% Lymph 15%
D1 14:15	BMP1: Na 141 K 5.1Cl 106 CO2 10 Glu 169 BUN 16 Cr 0.89	D1: 14:36	BMP1: Na 139 K 3.5Cl 108 CO2 24 Glu 111 BUN 16 Cr 0.77
D1 16:31	BMP2: Na 134 K 4.6Cl 102 CO2 14 Glu 385 BUN 18 Cr 1.0	D2 04:57	BMP2: Na 139 K 3.5Cl 104 CO2 22 Glu 99 BUN 9 Cr 0.63
D1 15:14	CT and CT angiogram chest: No PE. Extensive ground glass patchy opacities throughout the lung fields bilaterally; multiple bilateral rib fracture deformities; wall thickening of small bowel loops.	D1: 14:56	CT angiogram head and neck: Bovine arch; patent PICA origins. Patent vertebral arteries. Calcified plaque in the right carotid bifurcation and both internal carotid artery (ICA) siphons. Luminal irregularity in the cervical segments of right greater than left ICAs suggestive of artifact, remote trauma or artifact. OU
D2 16:40	CT chest: bilateral ground glass vs alveolar edema infiltrates improved. Small bilateral pleural effusions. Body wall anasarca. CAD and diffuse atherosclerosis; OU		
D1 14:15	Troponin I − 1 = 0.03	D1 14:36	Troponin I − 1 = 0.11
D1 16:31	Troponin I − 2 = 0.50	D1 18:25	Troponin I − 2 = 0.93
D1 18:29	Troponin I − 3 = 0.90	D1 21:30	Troponin I − 3 = 1.45
D1 21:18	Troponin I − 4 = 1.29	D2 01:48	Troponin I − 4 = 1.56
D2 00:15	Troponin I − 5 = 1.52	D2 04:57	Troponin I − 5 = 1.43
D2 03:26	Troponin I − 6 = 1.36 Units: ng/mL; Reference range < 0.03	ND	ND Units: ng/mL; Reference range < 0.03
D1 13:58	Lactic acid 1: 18.0	ND	ND
D1 14:15	Lactic acid 2: 17.0		
D1 16:31	Lactic acid 3: 6.6. Units: mm/L: Reference range = 0.5–2.0		
D1: 21:23	EEG: Generalized EEG suppression (<10 **μ**V)	D2 09:59	EEG: Normal EEG
D1-D3	cEEG: Severe suppression with emergence of a burst suppression pattern		
MPTA	Duloxetine 60 mg by mouth daily, Pramipexole 2.5 mg by mouth at bedtime.	MPTA	Duloxetine 60 mg by mouth daily, Pramipexole 2.5 mg by mouth at bedtime.
PMH	Obesity, HTN, hyperlipidemia, depression, restless leg syndrome and atrial fibrillation	PMH	Obesity, HTN, hyperlipidemia, depression and restless leg syndrome.

A subsequent chart review showed that sixteen months earlier she had been transported to the ED after coworkers heard a yelp and uninterpretable noises emanating from the office bathroom. After forcing the door open, EMTs found her on the floor, appearing combative and unable to speak or to follow commands. She was tachycardic, hypertensive and tachypneic. Her coworkers reported that she had acted normally that morning around the office. Given her sudden symptoms, she was triaged as a code stroke, but her mental status and language ability improved en route to the hospital where a stroke neurologist documented an initial score of zero on the National Institutes of Health Stroke Scale. She had no recollection of the event. Curiously, at that time, too, her workup demonstrated mild troponin I (Fig. 2) and peripheral leukocyte elevations (Table 1) that closely resembled those of her current hospitalization. In addition, the MRI of the brain during that admission also demonstrated an acute right cerebellar ischemic infarction in the same vascular territory (Fig. 1 F-G). A cardiac event monitor placed at discharge demonstrated several runs of atrial fibrillation within ten days. She was prescribed apixaban for this condition, but she did not take it. There was no evidence of infection or myocardial ischemia during either hospitalization but a CT of the chest and a CT angiogram of the head and neck, performed during her earlier hospitalization, showed diffuse coronary and carotid artery atherosclerosis (Table 1). Relevant data for both hospitalizations are shown in Table 1.

## Discussion

To our knowledge, this is the first report of cerebellar ischemic infarctions occurring concurrently with GTCSs, one of which resulted in a near-SUDEP diagnosis. It is not possible to establish whether the patient’s ischemic infarctions provoked her seizures, or vice versa, or whether the strokes occurred completely independently from the GTCSs. Unlike acute cortical infarctions, acute cerebellar infarctions are not considered triggers for early epileptic convulsions.[4] Given the PAF diagnosed after her earlier hospitalization, her ischemic infarctions could also be explained by cardioembolisms occurring before or during the GTCSs. Alternatively, the vascular territory affected could have been susceptible to infarction due to hypoxemia during the GCTSs, or due to hypoperfusion during her cardiac arrest. In the setting of her cardiovascular risk factors, such susceptibility could result from focal atherosclerosis, or, alternatively, from a predisposition to vasospasm in the affected vessels. The latter could be induced by catecholamines released into circulation during GTCSs,[5] as could her troponin I and leukocyte elevations.[6], [7] Thus, the coexistence of acute ischemic infarction, troponin elevations and leukocytoses after each GTCS suggests that this patient experienced semiological, or stereotyped, ictal or *peri*-ictal sympathetic (i.e., catecholaminergic) responses, which might have played a role in her *peri*-ictal cardiac arrest.

Troponins levels in the peripheral blood can increase due to physiological and non-physiological conditions ranging from strenuous exercise to myocardial ischemia.[6] Interestingly, uncomplicated GTCSs monitored in an epilepsy monitoring unit (EMU) did not increase troponin levels,[8] but similar seizures in refractory epilepsy patients[9] and in elderly patients with cardiovascular risk factors seen in the ED, did.[10] Considering our patient’s unfavorable outcome, these data suggest a potential association between postictal troponin elevations and clinically severe GTCSs. A similar conclusion can be reached about frank postictal leukocytoses (i.e., leukocyte elevations beyond the normal range) as uncomplicated GTCSs monitored in EMUs lead to leukocyte elevations within the normal range,[7] while GTCSs seen in the ED often lead to frank leukocytoses, particularly in cases of status epilepticus,[11] compromised breathing,[12] and postictal pulmonary edema.[13] Interestingly, in one recent study of aneurysmal subarachnoid hemorrhage,[14] both the troponin I and leukocyte count elevations were associated with the appearance of neurogenic pulmonary edema, which not only is frequently seen in SUDEP autopsies[15] but also appears to have contributed to this patient’s demise.

## Conclusion

This is the first report of apparently simultaneous and recurrent postictal cerebellar ischemic infarctions, troponin I elevations and mild leukocytoses in a near-SUDEP patient. Taken together, these findings suggest that stereotyped sympathetic responses might play a role in the pathophysiology of SUDEP, and that, in epilepsy patients with cardiovascular risk factors. postictal troponin I elevations and leukocytoses might signal an increased SUDEP risk.

## Ethical Statement

All the authors mentioned in the manuscript have agreed to be authors, have read and approved the manuscript, and have given consent for submission and subsequent publication of the manuscript.

## CRediT authorship contribution statement

**J.L. Vega:** Conceptualization, Writing - original draft, project supervision, Visualization. **A Carrasco:** Data Curation, Writing - review and editing, Visualization. **N Karim:** Investigation, Writing - review and editing. **M Stewart:** Investigation, Writing - review and editing. **W Bell:** Investigation, Writing - review and editing.

## Declaration of Competing Interest

The authors declare that they have no known competing financial interests or personal relationships that could have appeared to influence the work reported in this paper.

## References

[1] Nashef L., So E.L., Ryvlin P., Tomson T. (2012). Unifying the definitions of sudden unexpected death in epilepsy. Epilepsia.

[2] Devinsky O., Bundock E., Hesdorffer D., Donner E., Moseley B., Cihan E. (2018). Resolving ambiguities in SUDEP classification. Epilepsia.

[3] Devinsky O. (2011). Sudden, unexpected death in epilepsy. N Engl J Med.

[4] Lee S.H., Aw K.L., Banik S., Myint P.K. (2022). Post-stroke seizure risk prediction models: a systematic review and meta-analysis. Epileptic Disord.

[5] Devinsky O. (2004). Effects of Seizures on Autonomic and Cardiovascular Function. Epilepsy Curr.

[6] Agewall S., Giannitsis E., Jernberg T., Katus H. (2011). Troponin elevation in coronary vs. non-coronary disease. Eur Heart J.

[7] Vega J.L., Komisaruk B.R., Stewart M. (2022). Hiding in plain sight? A review of post-convulsive leukocyte elevations. Front Neurol.

[8] Woodruff B.K., Britton J.W., Tigaran S., Cascino G.D., Burritt M.F., McConnell J.P. (2003). Cardiac troponin levels following monitored epileptic seizures. Neurology.

[9] Nass R.D., Motloch L.J., Paar V., Lichtenauer M., Baumann J., Zur B. (2019). Blood markers of cardiac stress after generalized convulsive seizures. Epilepsia.

[10] Fawaz A., Nasreddine W., Makke Y., Atweh S., Wazne J., Arabi M. (2014). Association of cardiovascular risk factors and troponin elevation after generalized tonic-clonic seizures. Seizure.

[11] Aminoff M.J., Simon R.P. (1980). Status epilepticus. Causes, clinical features and consequences in 98 patients. Am J Med.

[12] Vega J.L., Emmady P., Roels C., Conforti J., Ramirez C., Dorak M.T. (2019). The Magnitude of Postconvulsive Leukocytosis Mirrors the Severity of Periconvulsive Respiratory Compromise: A Single Center Retrospective Study. Front Neurol.

[13] Romero-Osorio O.M., Abaunza-Camacho J.F. (2019). Sandoval-Briceno D. Rev Neurol.

[14] Nastasovic T., Milakovic B., Marinkovic J.E., Grujicic D., Stosic M. (2017). Could cardiac biomarkers predict neurogenic pulmonary edema in aneurysmal subarachnoid hemorrhage?. Acta Neurochir (Wien).

[15] Nascimento F.A., Tseng Z.H., Palmiere C., Maleszewski J.J., Shiomi T., McCrillis A. (2017). Pulmonary and cardiac pathology in sudden unexpected death in epilepsy (SUDEP). Epilepsy Behav.

